# Infodemia en tiempos de COVID-19

**DOI:** 10.26633/RPSP.2021.89

**Published:** 2021-07-06

**Authors:** Sebastián García-Saisó, Myrna Marti, Ian Brooks, Walter H. Curioso, Diego González, Victoria Malek, Felipe Mejía Medina, Carlene Radix, Daniel Otzoy, Soraya Zacarías, Eliane Pereira dos Santos, Marcelo D’Agostino

**Affiliations:** 1 Organización Panamericana de la Salud Washington D.C. Estados Unidos de América Organización Panamericana de la Salud, Washington D.C., Estados Unidos de América; 2 Universidad de Illinois Urbana-Champaign Estados Unidos de América Universidad de Illinois, Urbana-Champaign, Estados Unidos de América.; 3 Universidad Continental Lima Perú Universidad Continental, Lima, Perú.; 4 Organización Panamericana de la Salud San Pablo Brasil Organización Panamericana de la Salud, San Pablo, Brasil.; 5 Organización de Estados del Caribe Oriental Castries Santa Lucía Organización de Estados del Caribe Oriental, Castries, Santa Lucía.; 6 Red Centroamericana de Informática en Salud Ciudad de Guatemala Guatemala Red Centroamericana de Informática en Salud, Ciudad de Guatemala, Guatemala.; 7 Ministerio de Salud de Brasil Brasilia Brasil Ministerio de Salud de Brasil, Brasilia, Brasil.

El 15 de febrero de 2020, durante la Conferencia sobre Seguridad en Múnich (1), el director de la Organización Mundial de la Salud (OMS), el Dr. Tedros Adhanom Ghebreyesus, indicó que a la lucha contra la epidemia por la COVID-19 se le sumaba la lucha contra la “infodemia”, dando así inicio a una serie de acciones desde la OMS y otras organizaciones para enfrentar este desafío. Esta situación no es nueva, ya que casos similares han sucedido durante otras emergencias sanitarias, pero nunca antes con la magnitud actual, producto del aumento del uso de las aplicaciones digitales (2). En la era de la interdependencia digital, este fenómeno se amplifica debido a la convergencia del aumento en el acceso a los dispositivos móviles, el acceso a Internet y el uso de las redes sociales, que se propagan cada vez más lejos y más rápido, como un virus (3).

El término “infodemia” se deriva de la conjunción de los términos “epidemia” e “información” (4) y hace alusión a un exceso de información (veraz o no) que dificulta que las personas accedan a aquella proveniente de fuentes fiables y obtengan orientaciones válidas en momentos en que se hace más necesario para la toma de decisiones. La infodemia, además, hace referencia a un gran aumento del volumen de información relativa a un tema, que puede incrementarse de forma exponencial en muy poco tiempo por un incidente determinado, como es la pandemia de la enfermedad por el nuevo coronavirus (COVID-19) (3, 4). En esta situación aparece una mezcla de información científica y técnica con rumores, datos manipulados, falsos expertos, información incorrecta y noticias falsas y tendenciosas que dificultan el procesamiento y discernimiento por parte del receptor. Asimismo, el acceso a datos falsos o incorrectos produce distorsiones importantes en los modelos predictivos, lo cual afectando la planificación en los sistemas de salud y la toma de decisiones. Por ello, la comunidad científica está estudiando este fenómeno con el objetivo de entender sus patrones, estructuras y características, y así buscar mitigar sus consecuencias e intentar comprender el comportamiento de las personas frente a este fenómeno.

Por otra parte, la infodemia puede impactar de forma negativa en la salud y el bienestar, además de polarizar el debate público. Campañas en contra de las medidas de salud pública, datos epidemiológicos imprecisos o alterados y evidencia falsa o sesgada potencialmente podrían modificar el comportamiento de la población. Esto agrega una presión extra sobre el sistema de salud, ya que perjudica el alcance y la eficiencia de los diversos programas de intervención sanitaria.

Los principales factores que contribuyen al desarrollo de la infodemia se encuentran principalmente asociados a la falta de programas de alfabetización digital (5), que incluye: a) la dificultad de buscar, seleccionar, recomendar y diseminar críticamente datos e información confiables; b) la falta de criterios y herramientas para obtener información crítica en el formato y momento adecuados; y c) el desconocimiento en el uso y pertinencia de aplicaciones digitales en salud. Estos desafíos, que agregan una carga adicional a la pandemia, han acelerado la oportunidad de capacitar a la población e incorporar programas de formación continua de los trabajadores de la salud para desarrollarse funcionalmente en la era de la interdependencia digital (6).

Como miembros de la comunidad, todos tenemos un papel en la lucha contra la infodemia, el cual depende del lugar y el momento en el que nos encontramos frente a la información, incluidas acciones como: a) determinar si la información tiene sentido aun cuando provenga de una fuente segura y haya sido compartida anteriormente; b) confirmar la fuente; c) participar de manera responsable en las conversaciones sociales y, principalmente; d) ante la duda, tomar la decisión de no compartir información.

Como parte de la responsabilidad de la comunidad científica de profundizar el conocimiento sobre la infodemia, la Organización Panamericana de la Salud, en coordinación con la OMS, presenta este número especial con los estudios recientes más relevantes de la Región de las Américas, aportando orientaciones en un camino donde aún hay mucho por descubrir y por hacer.
